# Hydrogel Containing Anti-CD44-Labeled Microparticles, Guide Bone Tissue Formation in Osteochondral Defects in Rabbits

**DOI:** 10.3390/nano10081504

**Published:** 2020-07-31

**Authors:** Eva Filová, Zbyněk Tonar, Věra Lukášová, Matěj Buzgo, Andrej Litvinec, Michala Rampichová, Jiří Beznoska, Martin Plencner, Andrea Staffa, Jana Daňková, Miroslav Soural, Jiří Chvojka, Anna Malečková, Milena Králíčková, Evžen Amler

**Affiliations:** 1 Department of Tissue Engineering, Institute of Experimental Medicine of the Czech Academy of Science, Videnska 1083, 142 20 Prague 4, Czech Republic; eva.filova@iem.cas.cz (E.F.); matej@inocure.cz (M.B.); andrej.litvinec@iem.cas.cz (A.L.); michala.rampichova@iem.cas.cz (M.R.); martin.plencner@gmail.com (M.P.); andrea_mickova@labdemo.cz (A.S.); jana_dankova@labdemo.cz (J.D.); evzen.amler@lfmotol.cuni.cz (E.A.); 2 Institute of Biophysics, 2nd Faculty of Medicine, Charles University, V Uvalu 84, 150 06 Prague 5, Czech Republic; 3 Institute of Histology and Embryology and Biomedical Center, Faculty of Medicine in Pilsen, Charles University in Prague, Husova 3, 305 06 Pilsen, Czech Republic; zbynek.tonar@lfp.cuni.cz (Z.T.); anna.maleckova@lfp.cuni.cz (A.M.); milena.kralickova@lfp.cuni.cz (M.K.); 4 Hospital of Rudolfa and Stefanie, a. s., Máchova 400, 256 30 Benešov, Czech Republic; bezn@seznam.cz; 5 Department of Organic Chemistry, Faculty of Science, Palacky University, 17. listopadu 12, 771 46 Olomouc, Czech Republic; miroslav.soural@upol.cz; 6 Faculty of Textile Engineering, Technical University of Liberec, Studentská 2, 461 17 Liberec, Czech Republic; jiri.chvojka@tul.cz; 7 Student Science s.r.o., Národních Hrdinů 279, Dolní Počernice, 190 12 Prague, Czech Republic

**Keywords:** cartilage, CD44 antibody, collagen, fibrin, microparticles, poly-ε-caprolactone

## Abstract

Hydrogels are suitable for osteochondral defect regeneration as they mimic the viscoelastic environment of cartilage. However, their biomechanical properties are not sufficient to withstand high mechanical forces. Therefore, we have prepared electrospun poly-ε-caprolactone-chitosan (PCL-chit) and poly(ethylene oxide)-chitosan (PEO-chit) nanofibers, and FTIR analysis confirmed successful blending of chitosan with other polymers. The biocompatibility of PCL-chit and PEO-chit scaffolds was tested; fibrochondrocytes and chondrocytes seeded on PCL-chit showed superior metabolic activity. The PCL-chit nanofibers were cryogenically grinded into microparticles (mean size of about 500 µm) and further modified by polyethylene glycol–biotin in order to bind the anti-CD44 antibody, a glycoprotein interacting with hyaluronic acid (PCL-chit-PEGb-antiCD44). The PCL-chit or PCL-chit-PEGb-antiCD44 microparticles were mixed with a composite gel (collagen/fibrin/platelet rich plasma) to improve its biomechanical properties. The storage modulus was higher in the composite gel with microparticles compared to fibrin. The E_loss_ of the composite gel and fibrin was higher than that of the composite gel with microparticles. The composite gel either with or without microparticles was further tested in vivo in a model of osteochondral defects in rabbits. PCL-chit-PEGb-antiCD44 significantly enhanced osteogenic regeneration, mainly by desmogenous ossification, but decreased chondrogenic differentiation in the defects. PCL-chit-PEGb showed a more homogeneous distribution of hyaline cartilage and enhanced hyaline cartilage differentiation.

## 1. Introduction

The healing of osteochondral defects is limited and is accompanied by the ingrowth of fibrous tissue into the defect. However, the formed fibrocartilage cannot withstand the mechanical loading applied on the joint for an extended period; therefore, in non-treated defects, the degeneration symptoms progress. Standard surgical techniques, such as subchondral drilling or microfractures, are based on perforating the bone plate, which leads to bleeding and the migration of stem cells from the bone marrow into the defect. These techniques may decrease symptoms, but are not able to restore physiological hyaline cartilage [1,2]. The improved process of cartilage regeneration was introduced using autologous chondrocytes-seeded biomaterials as implants [3,4,5]. The limitations of chondrocytes implantation include invasive surgery for cartilage harvesting, the risk of dedifferentiation during in vitro cultivation, decreased quality of chondrocytes during ageing and the quality of chondrocytes which depend on the health of the donor.

Alternative methods that can promote physiological healing of osteochondral defects have been tested both in vitro and *in vivo.* Apart from chondrocytes [6,7], mesenchymal stem cells (MSC) have also been tested [8,9,10] after seeding in three-dimensional (3D) scaffolds, such as collagen [11], fibrin [12,13] and hyaluronan [14]. The crucial properties of the scaffolds, in order to promote cell migration, growth and differentiation, are contained in the scaffold composition, 3D structure, hydrogel-like structure or porosity, and the degradation rate. In addition, the scaffolds should have adequate mechanical properties. In our previous study we showed that polyvinyl alcohol (PVA) nanofibers embedded either in a composite collagen/hyaluronate/fibrin gel or fibrin alone showed increased Young’s modulus compared to composite gel or fibrin without nanofibers. (In the present study we concentrated on composite scaffolds with natural sources of growth factors from the present platelets.) Similarly, poly-ε-caprolactone (PCL) nanofibers embedded in a collagen/hydroxyapatite foam significantly improved the elastic modulus of the scaffolds [15].

A comparison of a cell-seeded scaffold with a cell-free scaffold showed a superior regeneration on the cell-seeded scaffolds, regardless of the presence of growth factors in the scaffold [5,11]. Although cell-seeded scaffolds show better results, there are many limitations connected with this procedure, such as an extra surgical procedure, donor side morbidity and inflammation or ex vivo expansion of aspirated cells. Thus, the novel attitude includes the cell-free scaffold implantation into an osteochondral defect in which the scaffold is able to enhance cell migration from the bone marrow into the scaffold and to stimulate MSC differentiation into chondrocytes. These scaffolds could be modified, e.g., by the incorporation of growth factors that enhance the stem cell migration into the scaffold and improve stem cell differentiation or other present bioactive chemicals that may have other functions, such as an anti-inflammatory or an immunomodulation effect [13,16].

Wang et al. used cell-free collagen scaffolds to heal osteochondral defects in rabbits. The mechanical properties of collagen were improved by adding polyacrylic acid grafted with the anti-inflammatory drug resveratrol [16]. In our previous study, the cell-free scaffold consisting of type I collagen/hyaluronan/fibrin was successfully tested on osteochondral defect healing in rabbits. The composite scaffolds contained a mixture of growth factors which stimulated chondrogenic differentiation of migrated MSCs and production of fibrocartilage after 6 weeks [13]. The same composite scaffold with growth factors was tested in miniature pigs. After 12 weeks the non-seeded scaffolds showed inferior cartilage regeneration compared to chondrocyte-seeded scaffold; the defects were filled mainly with fibrocartilage [5]. In the following study, the cell-free scaffold consisting of type I collagen/hyaluronan/fibrin was mixed with PVA nanofibers in order to improve the mechanical properties. Moreover, the PVA nanofibers were enriched with liposomes, basic fibroblast growth factor (bFGF) and insulin. Growth factor-enriched nanofibers were cut and mixed with a composite type I collagen/hyaluronan/fibrin gel and implanted into the load-bearing part of the femoral condyle. After 12 weeks, the cell-free scaffolds supported formation of fibrocartilage and hyaline cartilage in the defects. The positive effect was probably caused by controlled release of growth factors from liposomes, which was detected during 10 days for insulin and during 18 days for bFGF [17].

As the osteochondral defect has distinct zones with diverse demands on the osteogenic and chondrogenic parts, creating the multilayered scaffold is beneficial. Levingstone et al. tested a multilayered scaffold consisting of a collagen type I/hydroxyapatite layer followed by a collagen type I/hyaluronic acid layer, and the final layer was from collagen type I and II [18]. In this study, we prepared microparticles (MPs) from a blend of poly-ε-caprolactone-chitosan (PCL-chit) nanofibers or poly(ethylene oxide)-chit (PEO-chit). Subsequently, PCL-chit nanofibers were covalently bound with polyethylenglycol–biotin (PEGb) and the anti-CD44 antibody. The prepared scaffolds were tested for their biocompatibility *in vitro*. The CD44 is a cell-surface glycoprotein interacting with hyaluronic acid (HA) and other ligands of extracellular matrix. The rationale was to attract mesenchymal cells with the CD44 receptors into the wound and to evaluate the effect of CD44 neutralization. Our aim was to evaluate the effects triggered by two promising nanofibrous scaffolds in osteochondral defect regeneration in a rabbit model. Both PCL-chit and PCL-chit-PEGb-antiCD44 MPs were then put in the composite gel consisting of collagen/fibrin/platelet rich plasma (PRP) gel, and implanted into the osteochondral defects; they were evaluated after 6 weeks. In the present study we were concentrated on composite gel-containing natural sources of growth factors from the platelets. Moreover, the effect of the anti-CD44 antibody was studied in a gel without HA, as we expected the anti-CD44 antibody–HA interaction to affect cartilage regeneration. The characteristics of newly formed tissues were obtained through previously described standard histological techniques and differentiation of hyaline cartilage [13,19,20,21] and bone protein osteocalcin were traced and quantified.

## 2. Methods

### 2.1. Chemical Modification of Chitosan

#### 2.1.1. Preparation of the System of Chitosan-Spacer Arm (Chit-PEG-NH_2_)

**Figure 1:**
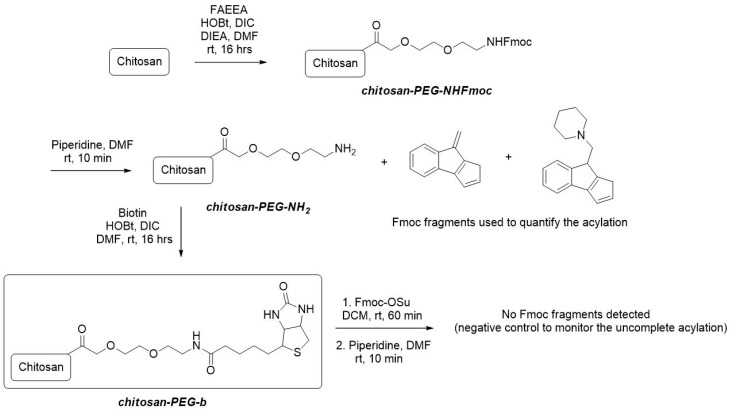
Synthetic approach to obtain the modified chitosan (chit-PEGb system).

A polypropylene fritted syringe was charged with 1 g of chitosan. A solution of 2-[2-(Fmoc-amino)ethoxy]ethoxy]acetic acid (FAEEA) (4 mM, 1540 mg), hydroxybenzotriazole (HOBt) (4 mM, 560 mg), N,N′-diisopropylcarbodiimide (DIC) (4 mM, 640 μL) and diisopropylethylamine (DIEA) (4 mM, 680 μL) in 10 mL dimethylformamide (DMF) was added to the syringe and it was shaken overnight. The content of the syringe was washed five times with DMF, three times with dichloromethane (DCM) and the resulting material was dried in a stream of nitrogen to give a white solid. Quantification: An amount of 10 mg of the solid product was treated with a solution of piperidine in DMF (5%, 1 mL) for 10 min and the solution was analyzed with liquid chromatograph–ultra violet–mass spectrophotometry (LC-UV-MS). The yield of acylation was calculated by analysis of Fmoc fragments from LC-UV traces at 300 nm with the use of Fmoc-Ala-OH as an external standard to give the loading 0.05 mM of FAEEA per 1 g of chitosan (Figure 1).

#### 2.1.2. Preparation of System Chitosan-Spacer Arm-Biotin (Chitosan-PEGb)

A polypropylene fritted syringe was charged with 1 g of chit-PEG-NH_2_, a solution of piperidine in DMF (5%, 10 mL) was added and the syringe was shaken for 30 min at room temperature. The content of the syringe was then washed 5 times with DMF. Biotin (1 mM, 244 mg) was dissolved in DMF (10 mL) at higher temperature (~80 °C). The solution was cooled to room temperature, HOBt (1 mM, 150 mg) and DIC (1 mM, 155 μL) were added, the resulting solution was added to the syringe and it was shaken overnight. The content of the syringe was then washed five times with DMF, three times with DCM and the resulting material was dried in a stream of nitrogen to give a white solid. Control: An amount of 10 mg of the product was shaken with a solution of Fmoc-OSu (100 mg) in DCM (1 mL) for 30 min. The solid was filtered and washed 5 times with DCM. The solid was then suspended in 5% piperidine in DMF (1 mL) and after 10 min, the solution was analyzed with liquid chromatography–mass spectrometry (LC-MS). No Fmoc fragments were detected.

### 2.2. Preparation of Nanofibers and Grinded Nanofibrous Microparticles

#### 2.2.1. Electrospinning of PCL-Chitosan and PCL-Chitosan-PEGb Nanofibers

PCL-chit (unmodified chitosan) and PCL-chit-PEGb (modified chitosan) nanofibers were prepared by electrospinning of 20% *w/v* poly-ε-caprolactone and 2% *w/v* chitosan dissolved in a mixture of glacial acetic acid and formic acid (7:3 (*v/v*)). The electrospinning was performed on a Nanospider NS500 device (Elmarco, Czech Republic) with maximal voltage up to 100 kV at room temperature. PEO-chit nanofibers were prepared from 1% (*w/v*) chitosan (medium-molecular weight, Sigma-Aldrich) and 0.5% PEO (MW 900 kDa, Sigma-Aldrich) dissolved in 90% (*v/v*) acetic acid and with the addition of 200 µM genipin as a crosslinker. Nanofibers were crosslinked at 55 °C for 48 h.

#### 2.2.2. Dry Cryogenic Grinding of Nanofibrous Mesh to Fibrous Microparticles

PCL-chit and PCL-chit-PEGb nanofibrous meshes were grinded by dry cryogenic grinding (Retsch CryoMill, Germany). The samples were cut into small pieces prior to grinding process. The nanofibers were placed into a 50 mL hardened steel grinding chamber and a 25 mm grinding ball was inserted into the chamber. The grinding was performed by 2 cycles of 30 s grinding with 10 s of homogenization. The grinding chamber was cooled by liquid nitrogen to maintain conditions during grinding under a glass transition temperature of PCL (−60 °C). The grinded particles were separated by a 700 µm sieve on a Retsch automatic siever.

#### 2.2.3. Modification of PCL-Chit-PEGb Microparticles by the Anti-CD44 Antibody

Avidin antibody conjugate was synthesized using EasyLink Avidin Conjugation kit (Abcam) following the manufacturer’s instructions. Briefly, 200 µg of avidin reagent was mixed with 200 μg of anti-CD44 antibody (Abcam, prod. No. ab119335). The antibody was mixed with PCL-chit-PEGb MPs (200 mg).

### 2.3. Characterization of Nanofibers and Nanofibrous Microparticles

#### 2.3.1. Binding of the HABA–Avidin Complex to Chitosan-PEGb

PEGb modified chitosan was tested for the binding of the 4′-hydroxyazobenzene-2-carboxylic acid (HABA)–avidin complex. The samples were incubated with 200 µL 80 mM of the HABA–avidin complex for 30 min. HABA is a weak avidin agonist and it is released from the complex in the presence of biotin. Displacement of HABA from the complex results in a decreased absorbance at 500 nm (Synergy H1, BioTek Instruments, Winooski, VT, USA). A decrease in the absorbance was calculated by the subtraction of sample absorbance and the absorbance of the HABA–avidin complex without incubation (control).

#### 2.3.2. Analysis of Morphology by Scanning Electron Microscopy

Nanofibers and MPs were characterized using scanning electron microscopy (SEM). Air-dried samples of electrospun nanofibers were mounted on aluminum stubs and sputter-coated with a layer of gold and analyzed by SEM (Tescan Vega 3, Brno, Czech Republic) at 10kV accelerating voltage.

#### 2.3.3. Fourier-Transformation Infrared Spectroscopy with Attenuated Total Reflection

The chemical composition of prepared materials was analyzed using Fourier-transformation infrared spectroscopy with attenuated total reflection (FTIR-ATR) spectroscopy. The samples of PCL-chit nanofibers, PCL nanofibers and chitosan powder were pelleted using a manual press. The pellets were analyzed using FTIR-ATR (IRAffinity-1, Shimadzu, MD, USA).

#### 2.3.4. Dynamic Laser Scattering

Size distribution of PCL-chit, PCL-chit-PEGb and PEO-chit MPs was measured by dynamic laser scattering (DLS). The MPs were dispersed in Tris buffer saline (TBS) with 1% Triton X-100. The measurement was performed on Mastersizer 3000 (Malvern, UK). The sample was added into the dispersing unit until optimal obscuration was achieved and the Mastersizer measured laser diffraction at 405 and 633 nm. The analysis was performed using Mie algorithm suitable for non-spherical particles. The particle distribution is represented as the volume density of each size fraction.

### 2.4. In Vitro Testing on Chondrocyte and Fibrochondrocyte Models

#### 2.4.1. Isolation of Chondrocytes and Fibrochondrocytes and Scaffold Seeding

Chondrocytes were isolated from the condyle of a pig’s femur obtained from a slaughter house (Jatky Český Brod, Český brod, Czech Republic). Fibrochondrocytes were isolated from meniscus of the same animal. We isolated and cultured the chondrocytes and fibrochondrocytes according to the previous protocol [5]. Briefly, the cartilage was cut into small pieces (approx. 1 × 1 mm), and incubated in a collagenase solution (0.3 PZ IU/mL, collagenase NB 4 G Serva Proved Grade, Serva, Heidelberg, Germany) in a humidified incubator (37 °C, 5% CO_2_) for 14 h. The cells were then centrifuged at 300× *g* for 5 min and seeded into culture flasks. The chondrocytes were cultured in a chondrogenic medium (Iscove’s modified Dulbecco’s medium) supplemented with 10% fetal bovine serum (FBS) (Sigma-Aldrich, Germany), penicillin/streptomycin (100 I.U./mL and 100 µg/mL, respectively, Sigma-Aldrich, USA), 4 mM L-glutamine (Gibco, UK), 100 nM dexamethasone (Dexamed; Medochemie, Czech Republic), 40 µg/mL L-ascorbic acid 2-phosphate (Sigma-Aldrich, Japan) and 1% insulin-transferrin-selenium-X (ITS–X, 10 µg/mL insulin, 5.5 mg/L transferrin, 6.7 µg/L sodium selenite, 2 mg/L ethanolamine, Gibco). Prior to cell seeding, both PEO-chit and PCL-chit nanofibers were cut into round patches 6 mm in diameter, and PCL-chit-PEGb-antiCD44 and PCL-chit-PEGb MPs were put into 96-well plates and sterilized using ethylene oxide. The samples were then seeded with 25 × 10^3^ porcine chondrocytes or fibrochondrocytes of the third passage. The samples with the cells were cultured in the chondrogenic medium. The culture medium was changed twice a week. We evaluated gene expression of the chondrogenic marker aggrecan in chondrocytes by qPCR. We proved that chondrocytes were able to maintain chondrogenic phenotype until passage 3, as shown in Figure S1.

#### 2.4.2. Cell Viability, Proliferation and Visualization

To determine the metabolic activity of the cells seeded on the prepared scaffolds, the MTS assay (CellTiter96^®^ AQueous One Solution Cell Proliferation Assay, Promega, WI, USA) was used on days 1, 8 and 15 of the experiment. Briefly, the scaffolds were transferred into new wells to prevent the cells becoming adhered to the tissue culture plastic. Subsequently, 100 μL of fresh media and 20 μL of the MTS substrate were added to each well. After 2 h incubation at 37 °C, 100 μL of the cultured solution was then transferred to a new clean well. The absorbance of the media was detected at 490 nm using a multi-mode microplate reader (Synergy HT, BioTek Instruments, Winooski, VT, USA). The background absorbance (690 nm) and the absorbance of the medium without cells were subtracted from the measured absorbance.

The proliferation of cells on the scaffolds was determined using a Quant-iT™ dsDNA Assay Kit (Thermo Fisher Scientific, Waltham, MA, USA) from the amount of DNA on days 1, 8 and 15. The scaffolds were put into a vial with 200 μL of cell lysis solution (0.2% *v/v* Triton X-100, 10 mM Tris (pH 7.0), and 1 mM ethylenediamine tetraacetic acid (EDTA)) and processed through 3 freeze/thaw cycles and roughly vortexed. A sample (10 µL) was mixed with 200 μL of reagent solution and fluorescence was measured using λexc = 485 nm, λem = 528 nm on the multi-mode microplate reader (Synergy HT, BioTek Instruments, Winooski, VT, USA). The DNA content was determined according to the calibration curve using the standards in the kit.

The cells on scaffolds or microfibers were fixed by frozen methanol (−20 °C) on days 1, 8 and 15, then washed twice with phosphate buffer saline (PBS). Cell membranes were stained with 1 µg/mL of DiOC6(3) (3,3′-dihexyloxacarbocyanine iodide; Invitrogen, Molecular Probes) for 45 min and rinsed with PBS. Cell nuclei were stained with 5 μg/mL propidium iodide (Sigma-Aldrich, USA) for 5 min, followed by rinsing with PBS. The cells were visualized on an Olympus FV10i confocal microscope (Olympus, Tokyo, Japan). λex = 488 and 560 nm and λem = 520 and 580 nm were used for DiOC6(3) and propidium iodide detection, respectively.

### 2.5. In Vivo Studies with Hydrogels Containing Nanofibrous Microparticles

#### 2.5.1. Preparation of Scaffolds for the in Vivo Experiment and Biomechanical Characterization of Hydrogels with Nanofibrous Microparticles

The graphical illustration of scaffold preparation is depicted in Figure S2. The scaffolds were prepared at 4 °C by mixing 6.4 µL of 5 mg/mL type I collagen (from calf hides, acid soluble, Symatese Biomatériaux, Chaponost, France) in 0.017 N acetic acid and neutralized with 2 M potassium hydroxide (KOH). Then, 35 µL of Iscove’s modified Dulbecco’s medium supplemented with penicillin and streptomycin, and 2.5 µg of MPs from either PCL-chit-PEGb or PCL-chit-PEGb-antiCD44, 25 µL of PRP, 100 µL of fibrinogen in aprotinin (including 91 mg/mL of fibrinogenum humanum and 3000 KIU/mL of aprotinium Tisseel Lyo 4 Kit, Baxter AG, Wien, Austria), and 100 µL of thrombin solution (500 IU/mL thrombinum humanum) in CaCl_2_ (40 µM/mL, Tisseel Lyo 4 Kit, Baxter AG, Wien, Austria) were added and stirred. Tromboconcentrate was obtained from the Hematology Service of the General Teaching Hospital, Prague, Czech Republic (volume 200 mL, thrombocyte concentration 200 × 10^9^) and prepared as previously described [22]. Briefly, tromboconcentrate was centrifuged (2250× *g*, 15 min), the supernatant was discarded and the resulting thrombocytes were washed three times in washing buffer as described by Baenziger [23]. Contaminating leukocytes and erythrocytes were removed by further centrifugation (120× *g*, 7 min). Thrombocytes were pelleted by centrifugation (2000× *g*, 15 min) and washed once and finally resuspended in buffer pH 7.5 (10^9^ mM NaCl, 4.3 mM K_2_HPO_4_, 16 mM Na_2_HPO_4_, 8.3 mM NaH_2_PO_4_ and 5.5 mM glucose) at a concentration of 120 × 10^6^/25 µL; they were then used for the preparation of one scaffold. The composite gel was formed at 37 °C. Subsequently, the culture medium was added and the scaffold was placed in an incubator with a humidified atmosphere, 5% CO_2_ at 37 °C for 1 day. The scaffolds contained either MPs from PCL-chit-PEGb (scaffold #1) or PCL-chit-PEGb-antiCD44 (scaffold #2).

The evaluation of biomechanical properties was based on resonance method according to Filova et al. [24] on the apparatus previously reported [25]. Briefly, the sample was connected to the weight on the top and to the frame at the bottom. The weight was connected with a calibrated spring. After a short impulse, the system of the spring, weight and samples oscillated. The real and imaginary part of the complex modulus of the sample was calculated from the frequency and damping coefficient. The preload of the samples was adjusted using a micrometer screw.

For self-oscillation L(t) = L_0_.e^−kt^.sin(ω.t) where L is the deformation. If we suppose the behavior of the sample obeys the Voigt model, the Newton coefficient (N) is: N = k ∙ M ∙ k, where M is the mass of the inertial body. Hooke’s coefficient is calculated: $H=M\cdot \omega^{2}+\frac{N^{2}}{4M}$. The storage modulus (ED) is: $E_{D}=\frac{H\cdot I}{A}$ where l is the length of the sample and A is the cross sectional area of the sample. The loss modulus (E_loss_) is thus: $E_{loss}=\frac{N\cdot I}{A}$.

#### 2.5.2. Implantation of the Scaffolds

For this study, twenty Chinchilla rabbits, five-months old (4.1 ± 0.6 kg), were used. Animal care was in compliance with the Act of the Czech National Convention for the Protection of Vertebrate Animals used for Experimental and other Scientific Purposes, Collection of laws No. 246/1992, including amendments on the Protection of Animals against Cruelty, and Public Notice of the Ministry of Agriculture of the Czech Republic, and Collection of laws No. 207/2004, on Keeping and Exploitation of Experimental Animals.

The operation was performed under general anesthesia (O_2_ and Isoflurane) after administration of diazepamum (Apaurin, 15 mg pro toto), ketamine (55 mg/kg s.c.) and xylazine (5 mg/kg s.c.). After preparing the operation field, the lateral arthrotomy of the right knee joint with medial luxation of the patella was performed. Scaffold #1 and scaffold #2 were implanted into the load bearing part of right femoral condyle of rabbits. A scaffold was introduced into the 5 mm deep circular defect with a diameter of 5 mm. The defects of the articular cartilage were filled with PCL-chit-PEGb MPs mixed with collagen/fibrin/PRP gel (scaffold #1, *n* = 7), or PCL-chit-PEGb-antiCD44 MPs mixed with collagen/fibrin/PRP gel (scaffold #2, *n* = 8) or were left untreated to heal spontaneously without a scaffold (control group, *n* = 5). The scaffolds were fixed in situ with a tissue adhesive Tisseel Lyo. All lesions were sutured in the followed layers: the joint theca, muscles and subcutis using absorbable material; the cutis with a non-absorbable material. The sutures in the cutis were removed 12 days after the operation. All the animals received preventive doses of antibiotics (Peni-Kel 300; 8000 international unit (I.U.) /kg blood weight (b.w.)) and analgesic (Metacam inj. ad us. vet.) by subcutaneous administration.

The samples were harvested after a healing period of six weeks following the formation of osteochondral defects sized 4.5 × 5 mm. All animals were sacrificed under general anesthesia by a lethal intravenous injection of T-61 six weeks after the scaffold implantation. The femoral condyles, including the site of the test item administration, were taken away and fixed in 10% phosphate buffered formaldehyde for histological examination.

#### 2.5.3. Histological Evaluation

All samples were decalcified for 8 weeks in a 12.5% solution of ethylenediaminetetraacetic acid (EDTA, Komplexon III p.a., Penta, Prague, Czech Republic) neutralized to pH = 7 by adding 1.25% (w/w) NaOH. After 8 weeks, EDTA was washed out from the samples using physiological solution for 24 h. Tissue blocks were cut in the center of the defect into two equal parts that were processed individually. Slices 5 µm thick of each sample were stained using different histological methods.

Preparations stained by hematoxylin and eosin (H&E) and green trichrome with Verhoeff’s hematoxylin were used for overall histology [26] (Figure S3). Picrosirius red (Direct Red 80, Sigma-Aldrich Aldrich, Munich, Germany) diluted in saturated picric acid solution was used to visualize the type I collagen using circularly polarized light [27] (Figure S3). A circular polarizing filter was crossed with a quarterwave λ/4 filter below the analyzer filter (U-GAN, Olympus, Tokyo, Japan) mounted on the Olympus CX41 microscope (Olympus, Tokyo, Japan). The presence of type II collagen was assessed in immunohistochemical sections using mouse monoclonal antibodies (clone II-II6B3-c, dilution 1:20, Developmental Studies Hybridoma Bank, Department of Biological Sciences, University of Iowa, Iowa City, IA, USA) (See Section 3.8.). To prove the differentiation of bone tissue, we performed an immunohistochemical detection of a bone protein osteocalcin that is positive in osteoblasts and bone matrix [28]. This was carried out using a monoclonal mouse anti-osteocalcin antibody (clone OCG3, dilution 1:200, Abcam plc, Cambridge, UK). Visualization of both immunohistochemical methods was based on diaminobenzidine (ImmPRESS antimouse Ig peroxidase polymer detection kit, Vector Laboratories, Burlingame, CA, USA) and the cell nuclei were counterstained with Gill’s hematoxylin. Acidic and neutral glycosaminoglycans were detected using a combination of alcian blue staining at pH 2.5 and Periodic Acid Schiff (PAS) reaction (Merck 101,647 Alcian blue and Merck 109,033 Schiff’s reagent, Darmstadt, Germany). Combining alcian blue with the PAS stain is a classical [29], but still very effective method [30] of demonstrating the glycosaminoglycans of the hyaline cartilage matrix (Figure S3). Alcian blue binds at pH 2.5 to the acidic glycosaminoglycans and stains them cyan, while PAS demonstrates neutral hexoses or the sialic acid and results in a magenta stain. The mixture of both types of glycosaminoglycans appear as purple.

From each sample, 20 slices (i.e., 10 slices from each half) representing the central part of the defect were stained by the alcian blue/PAS method and used for the quantification of hyaline cartilage found within the central area of the defect. From each sample, six more slices (three from each half) representing the central part of the defect were processed immunohistochemically for osteocalcin. Using a 10× objective, all the fields of view containing hyaline cartilage (on alcian blue/PAS sections) and osteocalcin-positive bone and osteoblasts (on immunohistochemically stained sections) were photographed. Volume estimates were obtained by the point counting method integrated in the Ellipse software (ViDiTo, Košice, SR). The points of a randomly positioned and calibrated testing grid were counted when hitting the hyaline cartilage or the osteocalcin-positive tissue. Since the area corresponding to each point as well as the section thickness was known due to the calibration, the volume of the hyaline cartilage and the volume of the osteocalcin-positive tissue were calculated according to the Cavalieri principle [31]. In order to distinguish the bone newly formed during the healing from the pre-existing bone on the bottom of the defect, only the osteocalcin-positive trabeculae with osteocalcin-positive osteoblasts on the surface not exceeding the size of the original defect were considered for the volume estimates. No correction for the tissue shrinkage was conducted. Therefore, the volume estimates do not represent the original in vivo volumes, but they may still be used for comparing the groups under study. The following criteria were applied when identifying the hyaline cartilage and other types of tissues filling the defect [5,19,21].

### 2.6. Statistics

Quantitative data are presented as mean values ± standard deviation (SD). The averaged values were determined from at least 3 independently prepared samples. The results were evaluated statistically using one-way analysis of variance and the Student–Newman–Keuls test (SigmaStat 12.0, Systat, San Jose, CA, USA). The data from the histological analysis were processed with the Statistica Base 9 (StatSoft, Inc., Tulsa, OK, USA). Spearman rank order correlation analysis was used to measure the statistical relationships between the variables. Kruskal–Wallis ANOVA and the Mann–Whitney U test were used for testing the equality of population medians between the groups under study. All the results were considered statistically significant if *p* was <0.05.

## 3. Results

### 3.1. Chemical Modification of Chitosan

The synthetic strategy for the modification of chitosan was analogical to the previously reported modification of PVA nanofibers [32] and is depicted in Figure 1. Firstly, chitosan was acylated with FAEEA to obtain chit-PEG-NHFmoc intermediate. The purpose of FAEEA was to generate the spacer arm between the molecule of chitosan and biotin to eliminate the potential steric repulsion of chitosan with avidin/streptavidin. The Fmoc protective group was applied for the quantification of the spacer arm loading. Its cleavage with piperidine provided UV-active Fmoc fragments detectable with the use of LC-UV analysis. With the use of Fmoc-Ala-OH as the external standard, the loading was calculated as 0.05 mM/g. The released chit-PEG-NH_2_ intermediate was subsequently acylated with biotin to give the target chit-PEGb system. The rate of biotinylation was evaluated with the Fmoc-OSu/piperidine test, which provided negative results, thus, indicating that the acylation of chit-PEG-NH_2_ intermediate was quantitative. For biological assays, chit-PEGb was finally freeze-dried to quantitatively eliminate all used chemical solvents.

### 3.2. Nanofiber Preparation and Characterization

**Figure 2:**
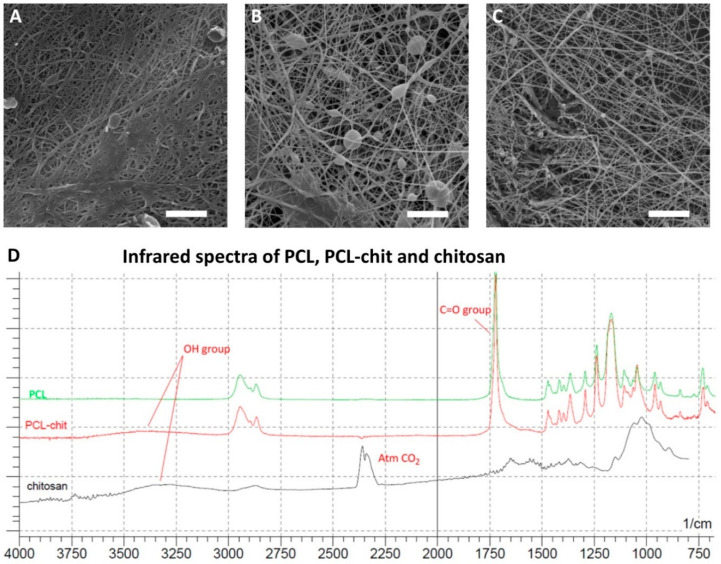
Morphology of nanofibers determined by SEM, poly-ε-caprolactone-chitosan (PCL-chit) (**A**), PCL-chit-PEGb (**B**) and poly(ethylene oxide)-chitosan (PEO-chit) (**C**). Characterization of PCL, PCL-chit and chitosan nanofibers by FTIR (**D**). Scale bar: 5 µm.

Nanofibers were prepared by needleless electrospinning using wire electrode (Figure 2A–C). The PCL-chit nanofibers had the mean fiber diameter 121 ± 59 nm. The fiber mesh contained only small pores with sizes up to 2 µm^2^. The fiber mesh contained minor fraction of non-fibrous defects. The samples prepared from modified PCL-chit-PEGb nanofibers showed a mean diameter of 123 ± 62 nm. However, the sample contained numerous globular defects. The size of pores was about 2 µm^2^. PEO-chit nanofibers had a mean diameter of 98 ± 48 nm. The mesh was formed by nanofibers with minimal defects. The mean pore size was up to 2 µm^2^. Thus, the results showed that the nanofibers PCL-chit and modified PCL-chit-PEGb had a similar morphology, fiber diameter (*p* = 0.934) and pore size (*p* = 0.375). The mean diameter of PEO-chit nanofibers differed for both PCL-chit and PCL-chit-PEGb significantly (*p* < 0.001). On the other hand, the pore size did not differ significantly.

Successful blending of chitosan with PCL was confirmed by FTIR spectroscopy (Figure 2D). The PCL showed typical resonance of the C=O group with a resonance frequency of 1750 cm^−1^ and the CH_2_ group of about 2750–3000 cm^−1^. The chitosan showed a peak at about 3250–3500 cm^−1^ corresponding to OH groups that were detected in the chitosan sample. The composite PCL-chit nanofibers contained both CO and OH groups typical of PCL and chitosan, respectively, thus confirming the successful incorporation of chitosan into the structure of PCL fibers.

### 3.3. Biological Evaluation of PCL-Chitosan Nanofibers

In order to evaluate the biocompatibility of scaffolds prepared from PCL-chit and PEO-chit, scaffolds were seeded by chondrocytes or fibrochondrocytes. In this study, PCL or PEO were blended with chitosan in order to enable the modification of the nanofibers with the anti-CD44 antibody.

**Figure 3:**
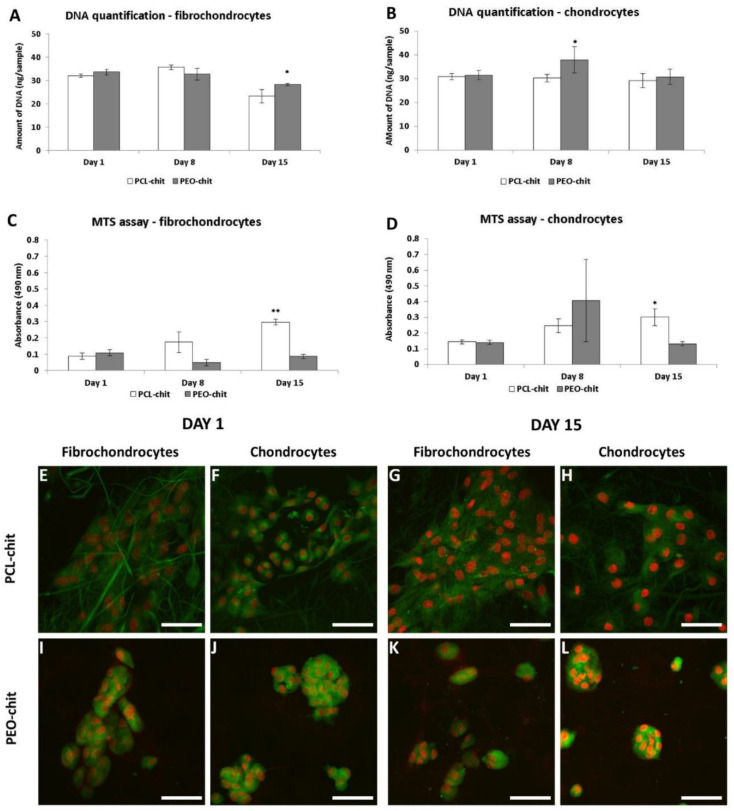
DNA quantification (**A**,**B**) and metabolic activity, measured by MTS assay (**C**,**D**) and visualization of fibrochondrocyte and chondrocytesadhesion and distribution on PCL-chit and PEO-chit scaffolds on days 1 and 15 using a confocal microscope (**E**–**L**). The values were compared statistically between the samples on the same day. Cell nuclei were stained using propidium iodide (red color) and cell internal membranes using DiOC3 (green color). Magnification: 600×; scale bar: 50 µm. Data are shown as mean ± standard deviation, * *p* < 0.05 and ** *p* < 0.001.

Quant-iT™ dsDNA Assay Kit was used for the quantification of DNA. The amount of DNA is an indicator of the cell number on the scaffolds. We observed a higher DNA content of fibrochondrocytes on PEO-chit scaffold on day 15 (Figure 3A). The amount of chondrocyte DNA (Figure 3B) on the PEO-chit scaffold was significantly higher compared to PCL-chit on day 8. MTS assay was used to test the metabolic activity of the cells. The PCL-chit scaffold stimulated the metabolic activity of fibrochondrocytes (Figure 3C) to a higher extent than PEO-chit. Fibrochondrocytes displayed a similar absorbance on both scaffolds on day 1, however, on day 15 a significantly higher absorbance was found on PCL-chit. The metabolic activity of cells on the PEO-chit scaffold decreased. Similarly, chondrocytes seeded on the PCL-chit (Figure 3D) scaffold displayed a significantly higher absorbance compared to the PEO-chit scaffold on day 15. From the proliferation point of view, we did not observe huge proliferation of the cells. However, the cells seeded on the PCL-chit scaffold remain viable throughout the whole experiment and showed an increasing tendency of metabolic activity. The results further showed that blending PEO with chitosan did not improve its properties. Therefore, for further modification with the anti-CD44 antibody, PCL-chit was chosen.

Confocal microscopy was utilized to visualize the adhesion and morphology of seeded cells (Figure 3E–L). On day 1, chondrocytes adhered on PCL-chit nanofibers in large groups. The cells spread more on PCL-chit than on PEO-chit scaffolds. On the contrary, both fibrochondrocytes and chondrocytes on the PEO-chit scaffold formed small groups containing small round or solitary cells.

### 3.4. Grinding of Nanofibers to Microparticles and Functionalization by Anti-CD44

**Figure 4:**
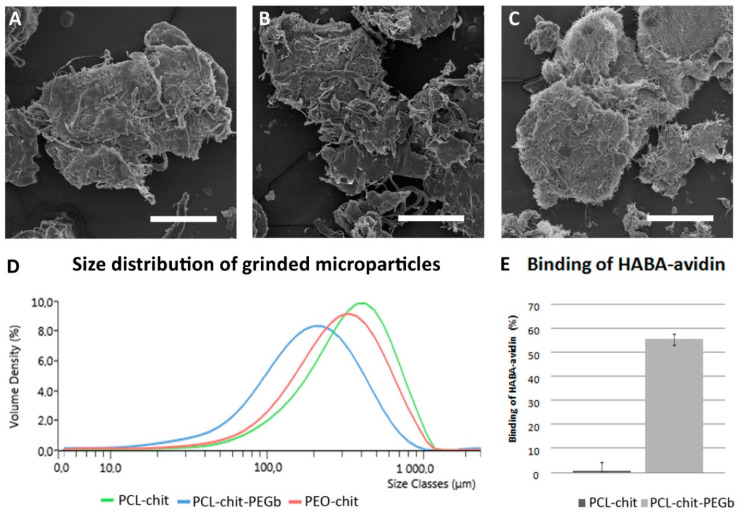
Morphology of grinded particles by SEM, PCL-chit (**A**), PCL-chit-PEGb (**B**) and PEO-chit (**C**). Distribution of microparticles (MPs) by DLS (**D**). Binding of the 4′-hydroxyazobenzene-2-carboxylic acid (HABA)–avidin complex to chitosan and to chit-PEGb (**E**). Scale bar: 100 µm.

The prepared nanofibers were disintegrated by a cryogenic grinding process in order to deliver an injectable nanofibrous scaffold. The process of cryogenic grinding was described recently by our group [33]. However, here we describe a modified approach based on a dry-cryogenic grinding method. The PCL-chit, PCL-chit-PEGb and PEO-chit nanofibers were cut into small pieces and grinded by an oscillation mill with liquid nitrogen cooling. Therefore, the process enabled efficient grinding of PCL under a glass transition temperature (−60 °C). The fibrous meshes become fragile and the process resulted in the formation of MPs with fibrous morphology. The morphology of particles is demonstrated in Figure 4A–C. A size analysis was performed by DLS (Figure 4D). The results showed that the PCL-chit MPs were larger and showed a mean size of about 500 µm. The PCL-chit-PEGb MPs had a mean size of about 200 µm. The PEO-chit MPs showed a mean size of about 400 µm. However, the size distribution of all MPs was wide and the grinding showed high polydispersity. For the later experiments, the MPs were sieved through a 700 µm sieve.

The objective of biotinylation of a chitosan based-nanofiber surface was to produce nanofibrous scaffold functionalized for a specific binding. The avidin–biotin complex is known as the strongest non-covalent interaction (dissociation constant Kd = 10^−15^ M). In addition, avidin with up to four binding sites for biotin, offers an exceptional binding affinity. The bond between biotin and avidin is formed very rapidly and, once formed, it is unaffected even by extreme environmental conditions [34]. The binding of HABA–avidin complex was tested. HABA is a weak agonist of avidin which is known to form a yellow-orange complex with avidin with a maximum absorption at λ = 500 nm. Biotin, a vitamin that has a very high affinity to avidin, is highly competitive with HABA, which results in its replacement and, consequently, the absorbance decreases. To observe specific avidin/biotin binding, PCL-chit and PCL-chit-PEGb were incubated with the HABA–avidin complex and the ratio of avidin bound to the nanofibers was calculated (Figure 4E). The PCL-chit bound only 0.7 ± 3.6% of the HABA–avidin complex. On the other hand, PCL-chit-PEGb bound 55 ± 2.3% of HABA–avidin and clearly showed that PCL-chit-PEGb nanofibers are able to bind avidin conjugates with high efficiency.

The MPs prepared from PCL-chit-PEGb were functionalized by the anti-CD44 neutralizing antibody. The anti-CD44 antibody was covalently conjugated to avidin and incubated with PCL-chit-PEGb MPs. The PCL-chit-PEGb-antiCD44 MPs were seeded with chondrocytes and fibrochondrocytes. Cell visualization by confocal microscopy was performed to verify cell adhesion and proliferation on MPs (Figure S1).

### 3.5. Biomechanical Characterization of Hydrogels

**Figure 5:**
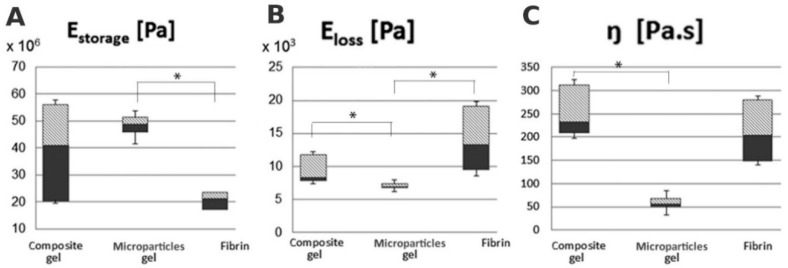
Viscoelastic properties of composite gel, microsphere gel (composite gel with PCL-Chit MPs) and fibrin were evaluated by dynamic tests, E_storage_ (**A**), E_loss_ (**B**) and viscosity (**C**) (* *p* < 0.05).

Storage modulus E_storage_ was significantly higher in the composite gel with MPs than in fibrin (Figure 5A). E_loss_ of both composite gel and fibrin was significantly higher than that of the composite gel with MPs (Figure 5B). Similarly, the composite gel showed a significantly higher viscosity than the composite gel with MPs.

### 3.6. Quantification of Hyaline Cartilage in the Center of the Bone Defect

**Figure 6:**
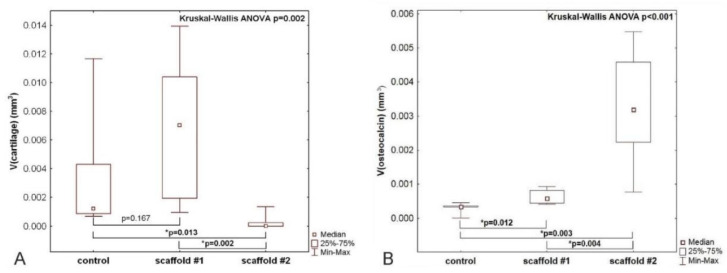
The volume of the newly formed hyaline cartilage (**A**) and osteocalcin-positive osteoblasts and bone matrix (**B**) within the center of the defect. A—The volume of the cartilage was greater in the scaffold #1 group (PCL-chit-PEGb MPs mixed with collagen/fibrin/PRP gel) than in the scaffold #2 group (PCL-chit-PEGb-antiCD44 MPs mixed with collagen/fibrin/PRP gel) (Mann–Whitney U test *p* = 0.002). The volume of cartilage was comparable in the scaffold #1 group and in the control group (untreated) and smaller in the scaffold #2 group than in the control group (*p* = 0.013). B—The volume of the osteocalcin-positive elements was greater in both the scaffold #1 (*p* = 0.012) and scaffold #2 (*p* = 0.003) groups when compared with the control group. The volume of osteocalcin-positive elements was greater in the scaffold #2 group than in the scaffold #1 group (*p* = 0.004).

The defects were prepared in a way that the subchondral bone was penetrated, enabling migration of progenitor cells into the implanted scaffold. The composite gel was implanted without further functionalization by cells, thus acting as a cell-free scaffold. A nonparametric analysis of variance revealed differences between the three experimental groups (*p* = 0.002), as shown in the graph in Figure 6A. The volume of newly formed hyaline cartilage in the central area of the defect was similar when comparing the group with scaffold #1 (PCL-chit-PEGb MPs mixed with collagen/fibrin/PRP gel) and the control group (untreated). The volume of the newly formed hyaline cartilage in the central area of the defect was greater in the scaffold #1 group than in the scaffold #2 group (PCL-chit-PEGb-antiCD44 MPs mixed with collagen/fibrin/PRP gel) (*p* = 0.002). The volume of newly formed cartilage was smaller in the scaffold #2 group than in the control group without a scaffold (*p* = 0.013).

### 3.7. Quantification of Osteocalcin-Positive Cells and Matrix in the Centre of the Defect

The volume of osteocalcin-positive cells and bone matrix had a negative medium correlation with the volume of cartilage within the same compartment of the healing defect (Spearman R = −0.52). Nonparametric analysis of variance revealed differences between the three experimental groups (*p* < 0.001). Volume of osteocalcin-positive elements in the central area of the defect was greater in both scaffold #1 (*p* = 0.012) and scaffold #2 (*p* = 0.003) groups when compared with the control group. The volume of osteocalcin-positive elements was greater in the scaffold #2 group than in the scaffold #1 group (*p* = 0.004), as shown in the graph in Figure 6B.

### 3.8. Distribution of Hyaline Cartilage, Bone Trabeculae and Qualitative Observation

**Figure 7:**
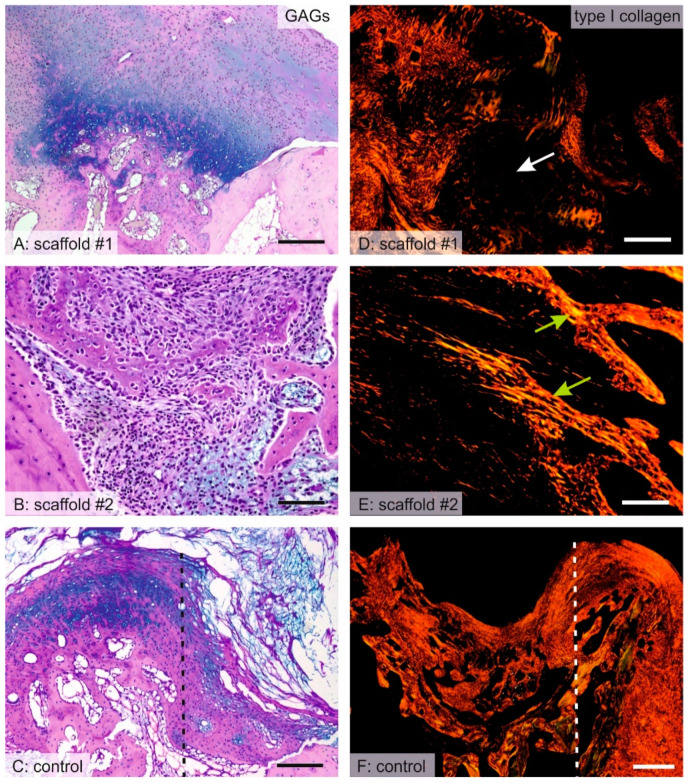
Comparison of the histology of healing with scaffold #1, scaffold #2, and without a scaffold. The microphotographs show either the central part of the defect (**A**–**C**,**E**) or the peripheral part of the defect (**C**,**F**). The border between the defect (C—right part of the image, F—left part of the image) and the original cartilage is marked by the dashed line. The alcian blue/PAS stain (**A**–**C**) demonstrated the glycosaminoglycans and the morphology of the samples. A—In samples with scaffold #1, a similar amount of hyaline cartilage was found when compared to the control samples (**C**). B—In samples with scaffold #2, desmogenous ossification was found on the bottom and on the sides of the defect. Picrosirius red (**D**–**F**) shows type I collagen fibers as red to yellow when observed in polarized light (right). D—Regions occupied by hyaline cartilage were negative for type I collagen and were dark (white arrow). E—The matrix of bone trabeculae found in the samples with scaffold #2 were positive for collagen I (green arrows). F—In control samples, large areas were filled by type I collagen-positive connective tissue. Scale bar: 200 μm (**A**,**D**); 100 μm (**B**,**C**,**E**); 500 μm (**F**).

**Figure 8:**
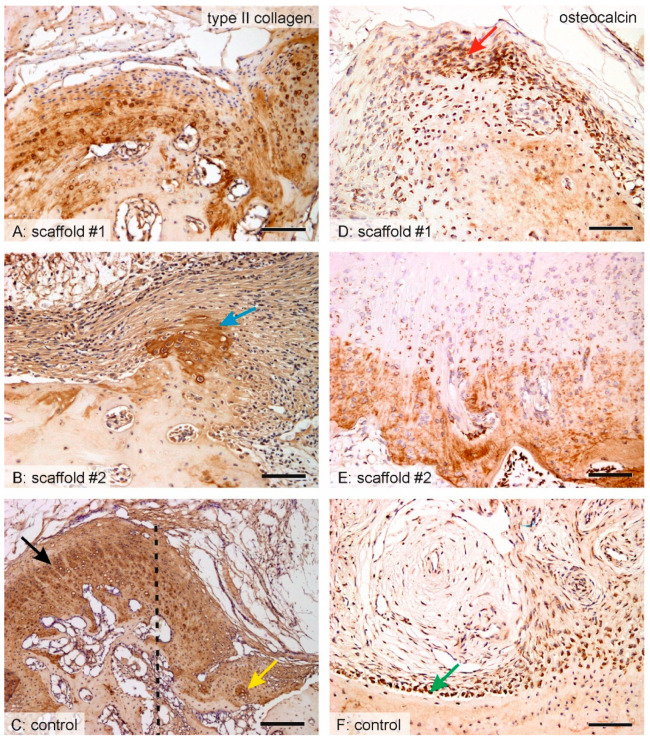
Comparing the histology of healing with scaffold #1, scaffold #2, and without a scaffold using type II collagen and osteocalcin immunohistochemistry. The microphotographs show the central part of the defect (**A**,**B**,**D**–**F**) or the peripheral part of the defect (**C**). A—In samples with scaffold #1, type II collagen-positive regions of hyaline cartilage were distributed in various regions of the defect, but mostly adjacent to the bone. D—In samples with scaffold #1, the surface of the bone was usually covered with osteocalcin-positive osteoblasts that were sometimes arranged in groups (red arrow). B—In samples with scaffold #2, only small islets of hyaline cartilage (blue arrow) were found. E—In samples with scaffold #2, the wall of the defect contained bone trabeculae with osteocalcin-positive bone matrix protruding into the defect and covered by osteoblasts. C—In control samples, the newly formed type II collagen-positive cartilage within the defect (yellow arrow) was only found close to the wound margins (dashed line, defect is on the right side of the image) and they were continuous with the articular cartilage outside the defect (black arrow). F—In control samples, most of the bone at the bottom and on the sides of the defect was covered by a single layer of osteocalcin-positive osteoblasts (green arrow). Immunohistochemistry for type II collagen (left) and osteocalcin (right) displayed a positive reaction in dark brown, counterstaining Gill’s hematoxylin. Scale bar: 100 μm (A, B, C right) and 200 μm (C left).

In scaffold #1, the center of the defect was mostly filled with granulation connective tissue, scaffold remnants, and islets of hyaline cartilage. The hyaline cartilage was present, even in the deep layers of the defects adjacent to the bone at the bottom and at the sides of the defect (Figure 7A and Figure 8A). In scaffold #2, hyaline cartilage was extremely rarely found (Figure 8B) and the defect was filled by remnants of the scaffold and granulation connective tissue with frequent inflammatory infiltration (Figure S4). At the bottom and on the lateral sides of the defect, the connective tissue bordered with newly forming bone trabeculae that originated from desmogeneous ossification (Figure 7B,E). The bone trabeculae were covered by osteoblasts and they had no connection to hyaline cartilage. The hyaline cartilage found in the control samples was found only at the border of the defects, growing towards the center of the defect from the margins and with the articular hyaline cartilage that was preserved (Figure 7C and Figure 8C). Osteocalcin-positive osteoblasts and bone matrix covered the walls of the defect in both scaffold #1 (Figure 8A) and scaffold #2 (Figure 8B), but the bone trabeculae were more branched and invaded the defect in scaffold #2 more so than in scaffold #1. The osteocalcin-positive tissue had a partial overlap with the calcifying hyaline cartilage, but most of the osteocalcin-positive bone tissue was independent of cartilaginous tissue, thus originating from desmogeneous ossification.

## 4. Discussion

The hydrogels provide a viscoelastic 3D environment stimulating chondrogenic differentiation of the MSCs and chondrocytes [35,36,37]. The synthetic or natural-derived polymers e.g., fibrin, gelatin, alginate, chitosan and HA have already been used for cartilage regeneration [12,38,39,40,41,42,43,44]. However, they possess low mechanical properties. On the other hand, the addition of woven or non-woven fibers, nano- and micro-fibers into the gel can improve the viscoelastic properties of the gel [45,46,47,48].

Electrospinning is a facile technique for the preparation of biocompatible materials supporting cell adhesion, proliferation and differentiation. Chitosan is a biocompatible and biodegradable polysaccharide [49]. A natural biopolymer, chitosan, was selected as a material for the production of nanofibers. Although chitosan solutions are highly viscous and difficult to electrospin, several publications have shown successful electrospinning of pure chitosan [50,51]. In this study, we have focused on the evaluation of chitosan blends. Chitosan was reported to be combined with a series of polymers including PVA [52], PEO [53,54,55], polyamide-6 [56], polyethylene terephthalates [57] and PCL [58]. The solution is typically dissolved in acetic solvents (e.g., acetic and formic acid) and shows antibacterial properties in combination with good biocompatibility [59].

In the present study, we prepared chitosan-based nanofibers by needleless electrospinning on a wire electrode. Needleless electrospinning has a higher production capacity, enabling the commercial production of nanofibers [60]. PEO-chit nanofibers prepared by blend electrospinning from 90% acetic acid solution resulted in the formation of homogenous nanofibers with a mean diameter of 98 ± 48 nm. The nanofibers contained a high concentration of chitosan (approximately 66%) and a minor component of PEO. In addition, the PEO-chit nanofibers were further stabilized by crosslinking. Genipin, a natural crosslinker, was utilized for the stabilization of PEO-chit nanofibers. The procedure of genipin crosslinking of chitosan films was previously reported by Jin et al. [61]. The second material was based on electrospinning of 22% (w/v) PCL with 2.5% (w/v) chitosan, resulting in the formation of nanofibers with a PCL-chit ratio of 9:1. The electrospinning was performed from acetic acid/formic acid system, previously described by Van der Schueren et al. [62]. The electrospinning process resulted in the formation of homogenous nanofibers with a mean diameter of 121 ± 51 nm. The morphology and diameter of nanofibers was similar to the results obtained by Van der Schueren who prepared nanofibers with a needle [62]; however, in this study a more productive needleless electrospinning electrode was utilized. The presence of chitosan in the PCL-chit nanofibers was confirmed by FTIR spectroscopy. The composite nanofibers showed a resonance of groups typical for both PCL and chitosan.

The biocompatibility of materials was evaluated on a model of fibrochondrocytes and chondrocytes. Chitosan was shown to efficiently stimulate chondrocyte adhesion and proliferation. Interestingly, the chondrocytes cultured on chitosan nanofibers, resembling a diameter of collagen fibrils (100–300 nm), showed the highest proliferation and chondrogenic extracellular matrix (ECM) production [63]. PCL is a biocompatible material which supports cell proliferation and is widely tested [64,65]; on the other hand, PEO is a hydrophilic polymer in which additives are beneficial in order to improve cell adhesion [66]. The goal of the in vitro experiment was to verify and compare if the prepared scaffolds are biocompatible and favor cell adhesion and proliferation; therefore, groups of solely PCL or PEO were not tested. In our experiment, fibrochondrocytes and chondrocytes seeded on PCL-chit and PEO-chit nanofibers showed good metabolic activity and a lower rate of proliferation. However, the metabolic activity was significantly higher on PCL-chit nanofibers. Confocal microscopy showed that chondrocytes formed small colonies on PEO-chit and did not allow cell spreading. The results of the experiment showed that both materials are promising for cartilage and meniscus tissue engineering, however, for the anti-CD44 antibody modification, PCL-chit with superior in vitro results was chosen.

The chitosan surface was further chemically modified to enable the binding of therapeutic proteins. In this study, we used the avidin-biotin system for the binding of the anti-CD44 antibody. The avidin–biotin system, for improved adhesion of cells, was reported for various cell types. Anamelechi et al. reported a system for the attachment of endothelial cells to 2D and 3D scaffolds [67]. In addition, Tsai et al. reported a system based on cell-culture plastic coating by the avidin–biotin system for the enhancement of chondrocyte adhesion [68,69]. Feng et al. prepared electrospun scaffolds with adsorbed avidin. Biotinylated Schwann cells showed improved adhesion to the avidin-modified scaffold and showed positive effect on proliferation and gene expression, demonstrating potential for neural tissue engineering [70].

CD44 is the main chondrocyte receptor for HA. The CD44 facilitates the interaction of chondrocytes with the supramolecular HA–proteoglycan complex. The CD44 therefore facilitates the formation of a gel-like structure around the chondrocytes. The elimination of these interactions results in decrease in aggrecan production and a loss of safranin O staining, and promotes chondrolysis cascade [71,72]. CD44 is present in various mammalian cells. Interestingly, cells can express CD44 in an active, an inducible, or an inactive state with respect to HA binding; such differences are cell specific and are reported to be related to posttranslational modifications. CD44 was found to be crucial for the maintenance of cartilage ECM homeostasis. CD44 is involved in internalization of HA, however, it also has an anabolic function. Interactions with intracellular proteins of the ERM family facilitate cell motility and migration [73]. The CD44 signalization pathway is complex and cell-type dependent. It is associated with the FAK/Src pathway, Rho/Rac pathway and Ras- mitogen-activated protein kinase (MAPK) pathway [74]. Therefore, the downstream CD44 signaling pathways are mainly associated with the regulation of cell proliferation and migration/adhesion. CD44 was shown to function in the migration of MSCs, triggered by HA as a chemokine. Zhu et al. showed impaired hyaluronan-induced migration by the neutralizing of CD44 [75]. In addition, CD44 was involved in the inhibition of catabolic MMP1/13 and ADAMTS4/5 expression [76,77]. Moreover, CD44 expressed on activated T cells binds HA, which was found to stimulate human T cell effector functions by CD3/TCR-mediated stimulation [78].

Ye et al. prepared scaffolds functionalized by CD90 antibody for the improvement of MSC adhesion. The avidin–biotin–CD90 system was utilized for the immobilization of cells onto the decellularized aortic valve. Immobilization by the antibody system showed tight immobilization even at high shear rates [79]. However, the mentioned studies utilize the common mechanism of function by conjugation of cells to antibodies prior to seeding and followed by adhesion enhancement by avidin bound on the scaffold. Yanada et al. developed CD44-functionalized magnetic beads for the labeling of MSCs. The MSCs were attracted to the site of chondral injury by the application of an external magnetic field [80]. Lin et al. prepared chitosan 2D and 3D scaffolds with bound avidin. The biotinylated anti-CD44 antibody recognized the receptor on chondrocytes. The system enabled the efficient improvement of cell adhesion mediated by the avidin–biotin interaction. In addition, the anti-CD44 antibody attachment to CD44 increased the mRNA expression of chondrogenic markers and glycosaminoglycans synthesis and enhanced cell proliferation and viability [81]. These observations are in accordance with our in vitro results. The CD44-modified MPs stimulated viability and proliferation of both fibrochondrocytes and chondrocytes (Figure S1).

However, for suggested microinvasive surgery, the nanofibrous mesh does not pose a suitable morphology. Therefore, the nanofibrous mesh was grinded into MPs by a dry cryogenic grinding method. Moreover, the MPs prepared from nanofibers have a high specific surface area. Thus, they can improve cell adhesion. The adhesion is also improved on the natural-derived polymers, e.g., collagen or chitosan, due to the present natural binding sites for cells. In our previous in vitro experiments, we used PCL MPs and PRP in a composite fibrin gel. We found that PRP improved MSC proliferation, and PCL MPs slowed down gel degradation compared to gel without MPs [47]. Moreover, PCL MPs are able to improve the biomechanical properties of the composite scaffolds, as was shown on the collagen–hydroxyapatite foams [15] and on the fibrin gel [17]. In addition, in situ formed chitosan-HA gel crosslinked with both genipin and β-glycerol phosphate significantly improved the biomechanical properties compared to the chitosan gel and stimulated chondrogenic regeneration in rats [82].

In order to mimic the natural microenvironment in the site of the defect, during the healing period, we mixed the MPs with the composite gel consisting of collagen, fibrin and PRP. Collagen is one of the most abundant fibrillar proteins presented in ECM. Fibrin gel is a natural polymer formed from fibrinogen and thrombin that has been successfully used in cartilage regeneration due to its natural net supporting cell adhesion, migration, and proliferation [83,84]. However, pure fibrin has some limitations, e.g., low both stiffness and viscoelasticity, and fast degradation. Therefore, it has been combined with HA, collagen, nanofibers, and with synthetic growth factors or platelet-derived growth factors (PDGF) [5,12,13,17,38]. The composite fibrin scaffolds provided improved viscoelastic properties and led to the regeneration of osteochondral defects. In order to deliver the natural mixture of growth factors that are present during the healing procedure we added the PRP into the composite gel. PRP is widely used in regenerative medicine for the healing of tendons, bones or cartilage [85,86,87,88].

The interaction of both MSCs and PRP seems to be important for bone regeneration. In clinical application, PRP enhanced bone defects healing. In osteochondral defects of the talus, patients that received surgery with PRP showed greater improvements than patients with PRP therapy but without surgery [89]. Prosecka et al. reported the highest volume of bone and uniform bone distribution in a collagen/hydroxyapatite/PCL scaffold modified with both PRP and autologous MSCs in rabbits. Significantly, a lower bone volume was observed in both the PRP group without cells, and the MSC-seeded group without PRP when compared to the PRP- and MSC-seeded groups. However, all mentioned groups showed significantly higher bone formation compared to the group with an empty defect [15]. PRP was found to stimulate an expression of chondrogenic genes such as collagen type II and aggrecan in chondrocytes of different origin and was also observed to decrease the expression of NF-κB and cyclooxygenase-2 [90].

We characterized the viscoelastic properties by the storage and loss moduli. E_storage_ is calculated from storage stiffness and represents the ability to store energy. E_loss_ is calculated from loss stiffness and characterizes the ability to dissipate energy [91]. Composite gel with MPs displayed higher E_storage_ compared to fibrin due to the MPs present in the composite gel. However, no difference in E_storage_ was found between the composite gel and the composite gel with MPs. Similarly, Young’s modulus in the collagen/hydroxyapatite scaffold enriched with PCL nanofibers was significantly increased compared to collagen/hydroxyapatite scaffolds [15]. Interestingly, previously we did not observe an increase in E_storage_ after adding of 10 or 20 wt% chitosan MPs in PCL foams (10 or 15 wt%). However, E_storage_ increased in foams prepared from a higher (15 wt%) PCL concentration compared to 10 wt% PCL regardless of the added chitosan MPs [24].

In our experiment, E_loss_ was lowest in the composite gel with MPs that correlated with viscosity measurement. The composite gel showed results similar to fibrin gel. On the other hand, chitosan MPs did not influence E_loss_ in PCL foams [24]. The collagen/chitosan foam showed decreased swelling compared to the pure collagen foam [92]. The addition of microfibrillar cellulose in the collagen/hydroxyapatite scaffold increased the compression strength in a dose-dependent manner. Biomechanical properties inversely correlated with water retention and the hygroscopicity of the scaffolds [93].

We previously tested the degradation of similar gel that was composed of fibrin/collagen type I/hyaluronate and porcine MSCs (2 × 10^6^/mL of the scaffold), and either PCL MPs, thrombocyte-rich solution (TRS) or both. We tested the weight differences between day 0 and day 7 and those between day 0 and day 14. The degradation assay showed the highest degradation of gel/TRS/PCL (46.2 ± 4.6 weight % on day 7 and 91.8 ± 3.3 weight % on day 14). The addition of PCL MPs into a composite gel decreased the degradation to 42.3 ± 2.3 weight % on day 7 and 84.4 ± 3.8 weight % on day 14. The slowest degradation was observed in the composite gel with PCL MPs: 37.6 ± 11.2 weight % on day 7 and 71.1 ± 1.9 weight % on day 14 [47].

PCL-chit (scaffold #1) or PCL-chit-PEGb-antiCD44 (scaffold #2) MPs were mixed with a composite gel consisting of collagen/fibrin/PRP, and implanted into the critical osteochondral defect in the rabbit model. The healing results showed that the PCL-chit MPs mixed with the composite gel enabled the formation of fibrous cartilage with hyaline cartilage on the basal part of the defect. Similarly, preferential cartilage formation on the basal parts and in parts adjacent to normal cartilage was found in the defects treated with non-seeded composite scaffolds from type I collagen/hyaluronate/fibrin containing growth factors and tested for osteochondral defect regeneration in rabbits and minipigs. Oppositely, in chondrocyte-seeded scaffolds, the cartilage formation was almost homogeneous in the defects [5,13]. This indicates differentiation into a chondrogenic phenotype in a bone/gel interface, however, further stimulation is necessary for the faster penetration of cells, even of superficial layers of the construct. Interestingly, PCL-chit-PEGb-antiCD44 MPs mixed with collagen/fibrin/PRP gel suppressed the chondrogenic differentiation of MSCs and led to an osteogenic phenotype. This scaffold significantly increased the amount of osteocalcin-positive tissue that refers to mineralized tissue and mature bone [94], which is synthesized by highly differentiated osteoblasts [95,96]. Alternatively, the cells formed bone trabeculae in the defect. In addition, the osteocalcin production was higher in this scaffold. The results indicate that the binding of anti-CD44 to the CD44-receptor resulted in impaired chondrogenic differentiation in vivo. A similar observation was reported by Zhu, who reported decreased rat MSC line Ap8c3-binding to both HA and fibronectin in the presence of the anti-CD44 antibody in a dose-dependent manner. The minimal efficient concentration was found to be 5 µL/mL for HA binding and 10 µL/mL for fibronectin binding. In addition, CD44−/− bone marrow derived MSC only poorly attached to HA even at a very low antiCD44 antibody amount (0.01 µL/mL), while CD44+/+ BMSCs decreased their adhesion to HA at 1 µL/mL anti-CD44 antibody [75]. These findings correspond to a decreased chondrogenic differentiation of the samples with PCL-chit-PEGb-antiCD44 composite gel.

PDGF was found to be a potent stimulator of rat MSC Ap8c3 cell synthesis of CD44. The values were significantly higher than the values found after the stimulation by transforming growth factor β (TGFβ), insulin growth factor-1 (IGF-I), bFGF and HA [75]. We have already found PDGF as the most abundant growth factor in platelets and observed its positive effect on cell adhesion, migration, proliferation, and osteochondral or bone regeneration [97,98,99]. Platelets were added into the scaffold as a source of growth factors that have been found to support bone or osteochondral regeneration [15,100,101,102]. The advantages are longer stability compared to synthetic growth factors and a possible autologous source of growth factors. It was reported that PDGF was able to stimulate rat MSC Ap8c3 cells and CD44+/+ BMSCs adhesion, but not CD44−/− BMSCs adhesion [75]. During our previous experiment in a minipig, we demonstrated the regeneration of osteochondral defects in cell-seeded scaffolds containing growth factors bFGF, IGF-I and TGFβ. The used growth factors supported MSC migration and differentiation into chondrocytes and hyaline or fibrocartilage production in a collagen/hyaluronate/fibrin scaffold [5]. Conversely, in the PCL-chit-PEGb-antiCD44 group, cell migration was decreased and chondrogenic differentiation was not observed. This finding is also supported by the observation of Shimizu et al. that CD44 plays an important role in adhesion and proliferation of multiple lineages, including T cells [103]. In addition, the uncoupling of chondrocytes from the ECM proteins, which is mediated through HA–CD44 interactions, may cause the induction of catabolic effects and may lead to the loss of cartilage homeostasis and induction of aggrecan and HA synthase [72].

Bone regeneration mainly occurs through Wnt/β-catenin, bone morphogenetic protein (BMP)/TGF-β, Notch, PI3K/Akt/mTOR, MAPK, PDGF, IGF, FGF, and Ca^2+^ pathways. Wnt/β-catenin signaling pathway is considered to be osteoinductive and mainly occurs in bone fractures. It regulates MSCs differentiation into osteoblasts [104]. Notch signaling through the activation of Notch receptors by their ligands has osteoinductive effects on osteoblasts [105]. The BMP/TGF-β pathway is necessary for osteogenesis in vitro and *in vivo*. Mainly BMP-2 is widely tested, but its dose and kinetics have to be optimized in vivo as side effects were often observed. BMP-2 and BMP-7 are strong activators of bone formation. They are pleiotropic proteins, meaning that they influence one or more signaling pathways which are not involved in bone regeneration [106]. bFGF promoted the expression of osteogenic markers such as Runx2, osteoprotegerin, p-Akt, and BMP-2 protein and enhanced osteogenesis on titanium surfaces via an activated PI3K/Akt signaling pathway [107]. IGF-I enhanced the osteogenic differentiation via the mTOR pathway [108].

## 5. Conclusions

PCL-chit nanofibers were successfully modified either with PEGb or the PEGb-antiCD44 system. The size distribution of MPs prepared by dry cryogenic grinding displayed a mean size of about 500 µm. The storage modulus E_storage_ of a composite gel with MPs was significantly higher compared to fibrin. The E_loss_ of both the composite gel and fibrin was significantly higher than that of the composite gel with MPs. Viscosity was significantly higher in the composite gel than that in the composite gel with MPs. The implantation of PCL-chit-PEGb MPs mixed with collagen/fibrin/PRP gel (scaffold #1) into the osteochondral defect of the rabbit did not result in a greater volume, but a more homogeneous distribution of hyaline cartilage that was newly formed within the center of the defect. The implantation of scaffold #1 resulted in a greater bone formation than in the controls. The implantation of the composite scaffold PCL-chit-PEGb-antiCD44 MPs mixed with collagen/fibrin/PRP gel (scaffold #2), resulted in more frequent inflammatory infiltration, and a smaller volume of hyaline cartilage. The desmogenous ossification induced within the defect was greater in scaffold #2 than in scaffold #1. We recommend scaffold #1 for further tests on the stimulation of hyaline cartilage differentiation and we recommend scaffold #2 for tests on the induction of desmogenous ossification.

## Supplementary Materials

The following are available online at https://www.mdpi.com/2079-4991/10/8/1504/s1, Figure S1: Visualization of fibrochondrocytes and chondrocytes adhesion and distribution on PCL-chit and PCL-chit-PEGb-antiCD44 MPs on days 8 and 15 using a confocal microscope. Cell nuclei were stained using propidium iodide (red color) and cell internal membranes using DiOC3 (green color). Magnification 600×, scale 50 µm. A—Relative expression of aggrecan, a chondrogenic marker, was analyzed by qPCR in order to characterize isolated chondrocytes. Chondrocytes seeded on tissue culture plastic in either chondrogenic (CH) or growth (G) media from passage 0 (P0) until passage 3 (P3) were evaluated. Statistical significance is shown above the columns (*p* < 0.05; * *p* < 0.01), Figure S2: Graphical visualization of scaffold preparation; Figure S3: Visualization of intact articular cartilage in five different histological staining methods (A–E). A—hematoxylin and eosin staining, B—green trichrome with Verhoeff’s hematoxylin, C—picrosirius red staining of collagen I fibers observed under circularly polarized light (yellow/red marking), D—immunohistochemical proof of collagen II (brown coloration), E—combination of alcian blue/PAS methods demonstrating acidic and neutral glycosaminoglycans. F—point counting method used for volume estimation. The primary criterion for hyaline cartilage identification displayed a positive result in alcian blue/PAS staining, and the secondary criteria were presence of collagen II and absence of collagen I fibers in picrosirius red staining. Scale bar: 100 µm (A–E) and 50 µm (F); Figure S4: Healing of the defect with scaffold #2. A—The defect was filled by remnants of the scaffold that is shown as eosinophilic amorphous matter (black arrow) occupying the center of the defect. B—The remnants of the scaffold were surrounded by granulation connective tissue with inflammatory infiltrates (yellow-dashed line) composed mostly of mononuclear infiltration with prevailing lymphocytes and macrophages (red arrows). Scale bar: 500 µm (A) and 50 µm (B).
