# Supplementary figures and images for: Hydrogel Containing Anti-CD44-Labeled Microparticles, Guide Bone Tissue Formation in Osteochondral Defects in Rabbits
Source: Nanomaterials (Basel). 2020 Jul 31;10(8):1504. doi: 10.3390/nano10081504 (PMC7466545; doi:10.3390/nano10081504)

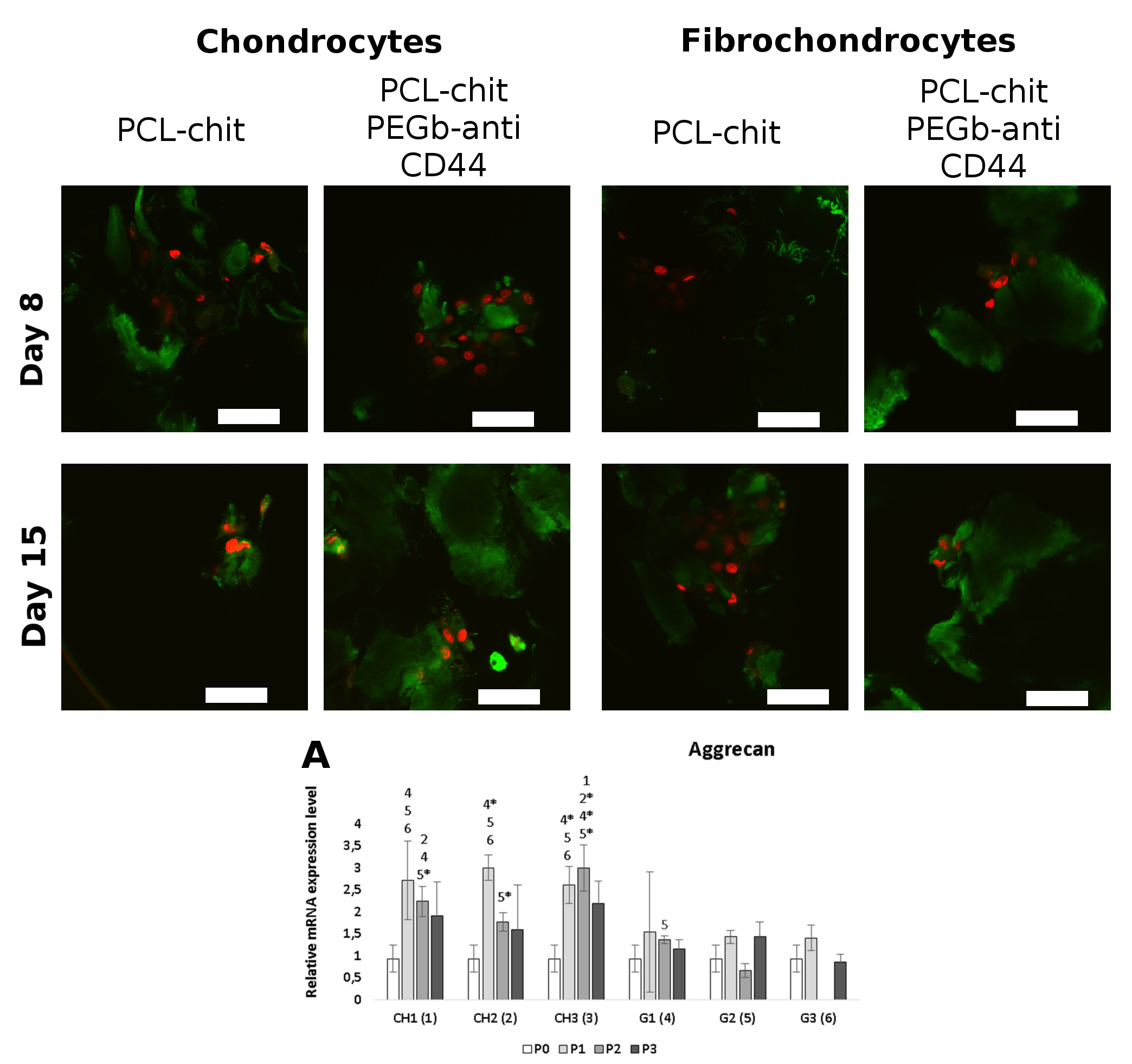

Supplement: Supplementary file 1 [file nanomaterials-10-01504-s001.zip › Figure S1 revised.jpeg]

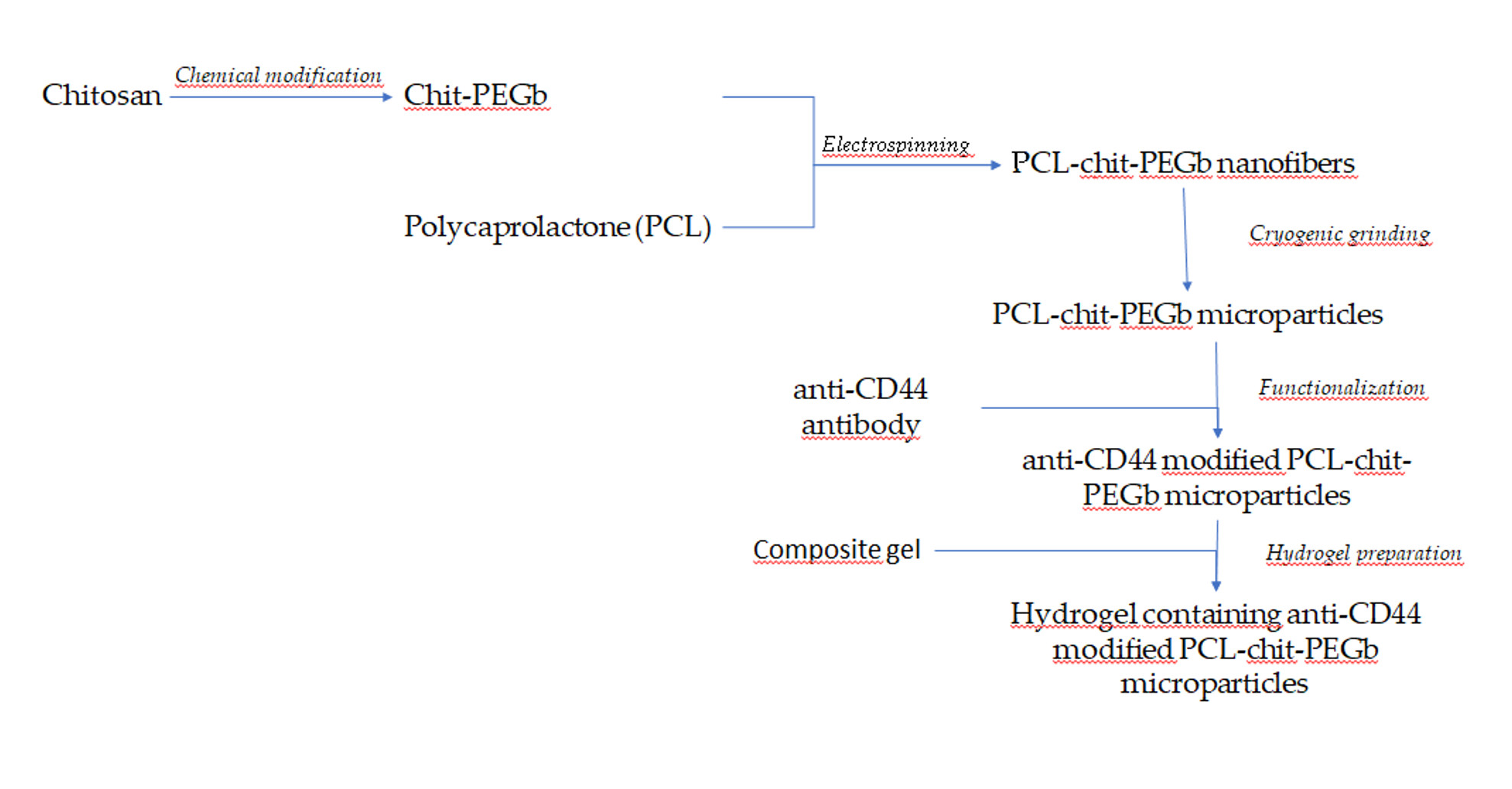

Supplement: Supplementary file 1 [file nanomaterials-10-01504-s001.zip › Figure S2 revised.jpeg]

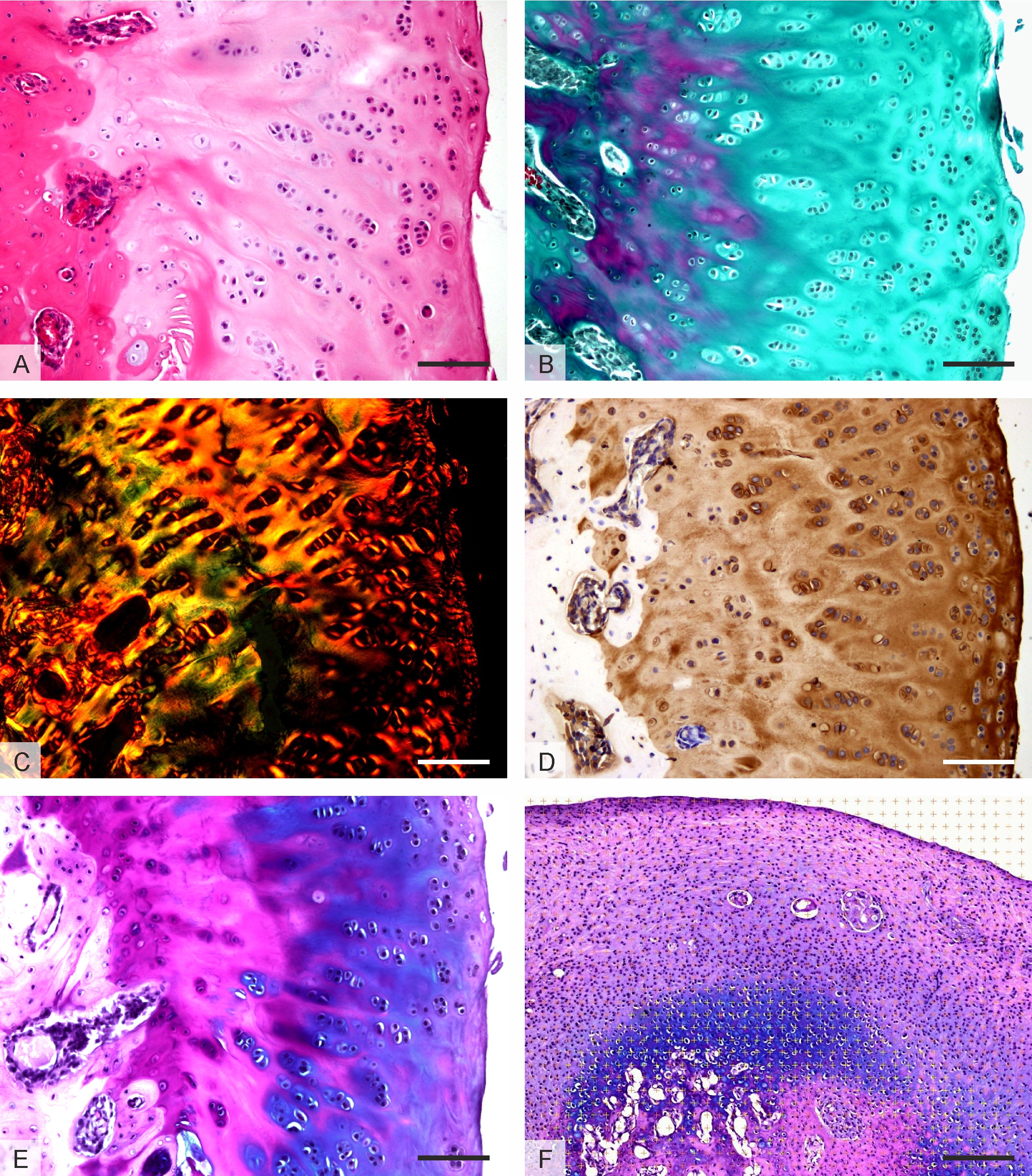

Supplement: Supplementary file 1 [file nanomaterials-10-01504-s001.zip › Figure S3.jpg]

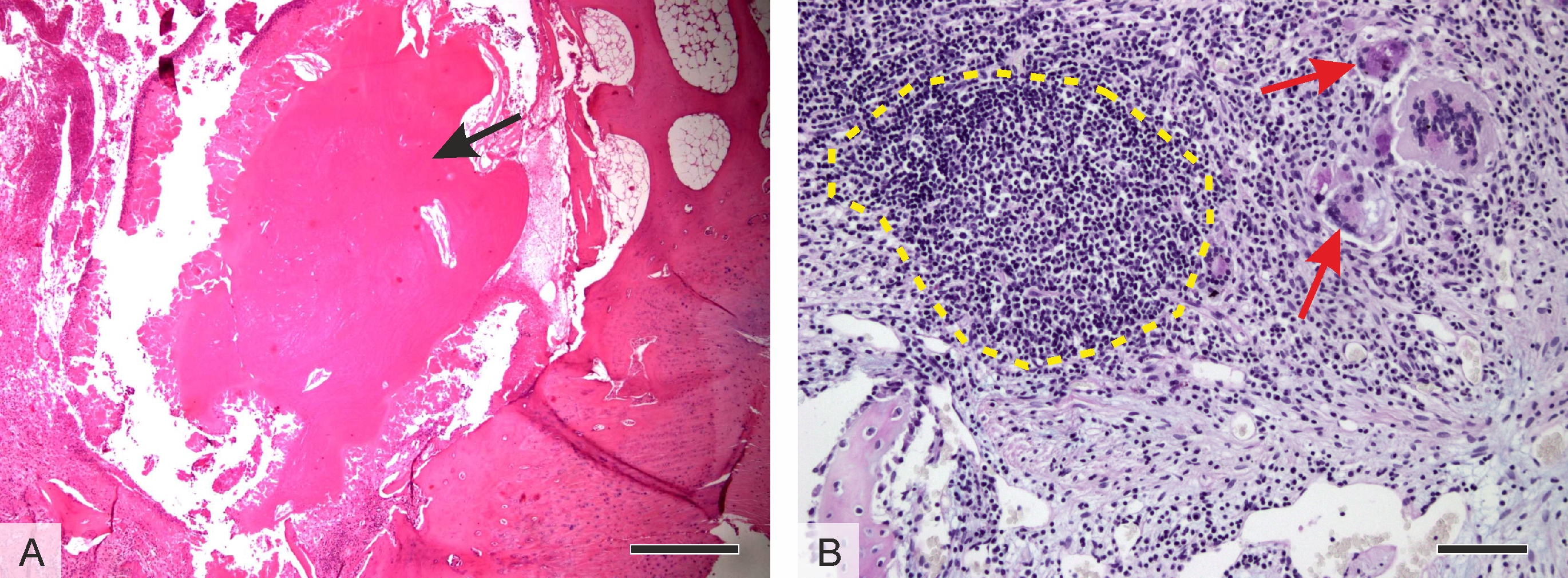

Supplement: Supplementary file 1 [file nanomaterials-10-01504-s001.zip › Figure S4.jpg]
